# A genetic approach of wine yeast fermentation capacity in nitrogen-starvation reveals the key role of nitrogen signaling

**DOI:** 10.1186/1471-2164-15-495

**Published:** 2014-06-19

**Authors:** Claire Brice, Isabelle Sanchez, Frédéric Bigey, Jean-Luc Legras, Bruno Blondin

**Affiliations:** INRA, UMR1083 Science pour l’Œnologie, 2 Place Viala, F-34060 Montpellier, France; Montpellier SupAgro, UMR1083 Science pour l’Œnologie, 2 Place Viala, F-34060 Montpellier, France; Université Montpellier 1, UMR1083 Science pour l’Œnologie, 2 Place Viala, F-34060 Montpellier, France

**Keywords:** Fermentation, Nitrogen, QTL mapping, *Saccharomyces cerevisiae*, TOR pathway, *MDS3*, *GCN1*, *ARG81*, *BIO3*

## Abstract

**Background:**

In conditions of nitrogen limitation, *Saccharomyces cerevisiae* strains differ in their fermentation capacities, due to differences in their nitrogen requirements. The mechanisms ensuring the maintenance of glycolytic flux in these conditions are unknown. We investigated the genetic basis of these differences, by studying quantitative trait loci (QTL) in a population of 133 individuals from the F2 segregant population generated from a cross between two strains with different nitrogen requirements for efficient fermentation.

**Results:**

By comparing two bulks of segregants with low and high nitrogen requirements, we detected four regions making a quantitative contribution to these traits. We identified four polymorphic genes, in three of these four regions, for which involvement in the phenotype was validated by hemizygote comparison. The functions of the four validated genes, *GCN1*, *MDS3*, *ARG81* and *BIO3,* relate to key roles in nitrogen metabolism and signaling, helping to maintain fermentation performance.

**Conclusions:**

This study reveals that differences in nitrogen requirement between yeast strains results from a complex allelic combination. The identification of three genes involved in sensing and signaling nitrogen and specially one from the TOR pathway as affecting nitrogen requirements suggests a role for this pathway in regulating the fermentation rate in starvation through unknown mechanisms linking nitrogen signaling to glycolytic flux.

**Electronic supplementary material:**

The online version of this article (doi: 10.1186/1471-2164-15-495) contains supplementary material, which is available to authorized users.

## Background

Yeast strains are the main microorganisms used in fermentation process. During wine fermentation, yeast and principally *Saccharomyces cerevisiae*, consumes the sugars found in the grapes musts and converts them into alcohol, carbon dioxide and secondary-ends products that contribute of wine character. To support yeast growth and enable it to perform these complex biochemical transformations a number of nutrients must be found in musts. The assimilable nitrogen is a key nutrient in the control of alcoholic fermentation and is consumed at the beginning of the process. Low nitrogen levels in musts may cause slow or stuck fermentation [1]. During fermentation, the ethanol concentration increases and can destabilize cell membranes in a manner that may result in an inability to take-up nitrogenous compounds from the must [2]. Alcoholic fermentation occurs principally in these stressed conditions, so the ability of the yeast to maintain high levels of fermentation activity in such conditions is crucial to the outcome of the alcoholic fermentation. Wine yeast strains differ in their capacities to carry out fermentation in conditions of nitrogen limitation. These differences have been described as reflecting differences in the nitrogen requirements of wine yeasts [3–5]. During alcoholic fermentation, such differences become visible after the yeast cells enter stationary phase in many cases though not all due to nitrogen starvation (i.e. in a complete exhaustion of assimilable nitrogen in must), through differences in the abilities of different strains to maintain a fermentation flux. Little is known about the mechanisms involved in controlling fermentation rate under nitrogen starvation and the limiting factors involved. It has been suggested that control over sugar transport capacity affects the rate of fermentation in nitrogen-starved cells. It has been shown that hexose carriers are targeted for degradation by endocytosis in such conditions, suggesting that carrier stability may affect the fermentation rate [6]. Yeasts are also subjected to the inhibitory effects of ethanol, which also decreases the fermentation rate [7]. Several studies have reported the genome-wide transcriptional response of yeast to nitrogen starvation in alcoholic fermentation conditions [4, 5, 8–12]. In a previous study, we showed that “low-nitrogen requirement strains” (LNR) strongly expressed biosynthetic genes, whereas “high-nitrogen requirement” (HNR) strains displayed a specific gene expression pattern, with the overexpression of stress genes. HNR strains seem to be more sensitive to nitrogen starvation, resulting in a stronger stress response and a lower fermentation rate. These differences in response are reminiscent of the differences in fermentation capacity described between sake and laboratory yeasts, resulting from differences in Rim15p function [13].

*Rim15p* is involved in nitrogen signaling downstream from TOR, which senses the nitrogen status of the cell and adapts cell metabolism to nutrient availability [14, 15]. These previous studies provided the first demonstration that nitrogen signaling could affect fermentation rate. Similar mechanisms may contribute to differences in the nitrogen requirements of wine yeasts and would be consistent with differences in transcriptional patterns. However, although physiological approaches have provided novel and relevant insight into the mechanisms associated with nitrogen requirements, the genetic variants underlying phenotypic differences have yet to be identified. The use of QTL analysis to identify the genes underlying this variation should improve our understanding of the mechanisms involved.

Nitrogen requirement is a quantitative trait. It is therefore possible to use a QTL approach to investigate the molecular basis of variation for this trait. QTL approaches have led to the detection of many genes, typically unlinked to each other [16]. In *Saccharomyces cerevisiae,* several QTL analyses have already been carried out, to identify genes involved in growth at high temperature [17, 18], sporulation [19–22], cell morphology [23], drug sensitivity [24], ethanol tolerance and growth [22, 23, 25–27], flocculation [28], wine aroma production [29], amino acids consumption [30], and to decipher regulatory network variations [31, 32]. These studies have shown some phenotypes to be highly complex. Some phenotypic variation may be accounted for by a single QTL with a major effect [33–36], or by several QTLs with minor effects [18, 37, 38]. It is thought that a large proportion of phenotypic variation is accounted for by QTL with smaller effect sizes [39–41]. The complexity of the phenotype is accounted for not only by the number of genes underlying the variation, but also by genetic interactions and epistasis between loci [42].

We investigated the genetic basis of the variability of nitrogen requirement, by establishing a genetic device based on bulk segregant analyses and using it to identify QTL. We identified four genes for which allelic variations between parental strains were associated with differences in fermentative activity in a medium in which nitrogen was limiting. Interestingly, three of these four genes were found to be involved in nitrogen sensing and signaling.

## Results

### Screening of parental strains and constitution of the study population

**Figure 1:**
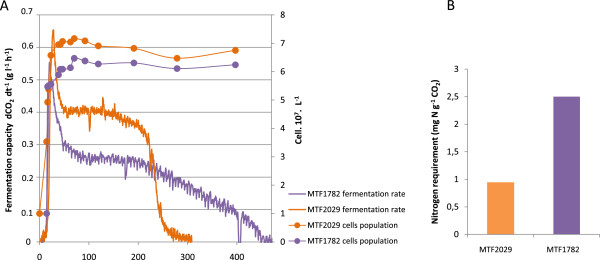
**Comparison between the two parental strains (MTF2029 and MTF1782).** Fermentation performances in nitrogen-deficient medium (SM100) **(A)**. Comparison of the nitrogen requirements during fermentation. Nitrogen requirement determined by the constant fermentation rate method **(B)**.

We investigated the genetic basis of variations of nitrogen requirements in wine yeast, in two *Saccharomyces cerevisiae* enological strains, MTF2029 and MTF1782, characterized in a previous study as displaying extreme differences in fermentation ability in musts in which nitrogen was limiting [5]. Constant fermentation rate (CFR) determinations [43] indicated that strain MTF2029 had a low nitrogen requirement (0.94 mg N g^−1^ CO_2_), whereas MTF1782 had a high nitrogen requirement (2.5 mg N g^−1^ CO_2_). The nitrogen requirements of these strains were directly correlated with their fermentation capacity in a nitrogen-deficient medium (Figure 1). At the start of the stationary phase, when cell growth had stopped, fermentation rate declined differently in these two strains. Strain MTF2029 maintained a high fermentation rate throughout stationary phase, whereas MTF1782 displayed a large drop in fermentation flux, resulting in a longer fermentation time. The nature of the nitrogen source contained in the synthetic medium must be taken in consideration because it can impact the fermentation efficiency. Moreover, the fermentation rate is positively correlated with both the total amount of assimilable nitrogen and the nitrogen uptake rate [12, 31]. Indeed, yeast can display differences in nitrogen compounds consumption [30, 44]. To avoid differences phenotypic caused by nitrogen source utilization, we ensured that the two parental strains show the same consumption profile for nitrogen sources. These differences in fermentation rate were not associated with differences in the use of available nitrogen sources, as both strains used all the assimilable nitrogen during the growth phase. These two strains were therefore considered relevant for use in studies of the genetic basis of variation in fermentation capacity in conditions of nitrogen deficiency. The choice of these strains was also based on their ability to sporulate (approximate of 30% for the two strains), because this characteristic is required for the construction of a recombined population. We have estimated a spore viability of 33% for MTF2029 strain and 44% for MTF1782 strain. A hybrid strain was obtained by crossing two parental haploid clones, 2029-C5 and 1782-B1, in which the *HO* gene had been inactivated. The hybrid strain had a fermentation profile intermediate between those of the two parental strains (Additional file 1: Figure S1). The zygote was allowed to sporulate and the F1 haploid clones obtained were used for a second round of crosses, generating 133 F2 haploid segregants.

### Phenotyping of the segregant population

**Figure 2:**
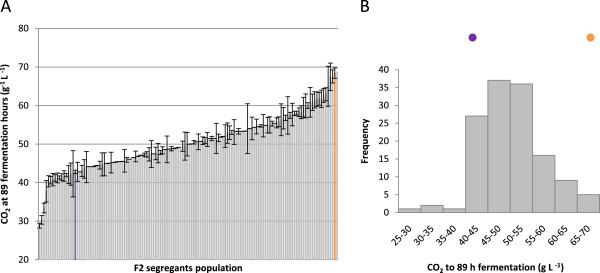
**Distribution of the amount of CO**_**2**_ **released at 89 hours in the segregating population in nitrogen-deficient medium (SM120).** Fermentation rate was measured as the amount of CO_2_ released at 28°C. Segregants were sorted according to cumulative CO_2_ release **(A)** and the fermentation rate histogram **(B)**. For both representation, purple and orange code represents the position of 1782-B1 and 2029-C5 parental strain, respectively.

**Figure 3:**
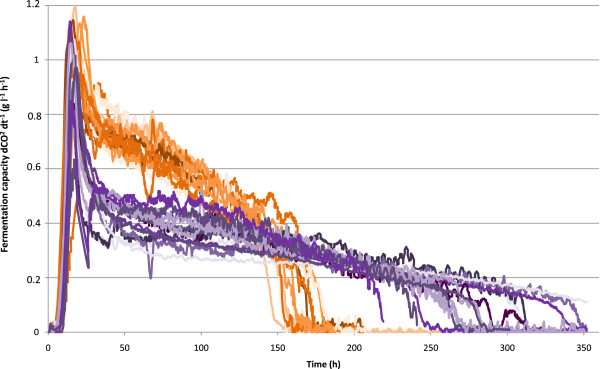
**Fermentation profiles obtained for strains from each bulk in nitrogen-deficient medium (SM120) at 28°C.** For fermentation representation, purple and orange code represents the HNR-bulk and the LNR-bulk, respectively.

We phenotyped the segregant population in nitrogen-limited fermentation conditions. We first determined the amount of CO_2_ produced after 89 hours of fermentation on a medium containing 120 mg of available nitrogen. We had previously checked that this measurement was representative of the fermentative capacity of the strain and correlated with nitrogen requirement determinations by the CFR procedure. The whole population was phenotyped in these conditions and the results are shown in Figure 2. This phenotypic trait displays a low transgression level with only 8 segregants in negative transgression corresponding at 6% of transgressive segregation in the progeny. More the control of variability revealed a heritability of 98% for the character indicating that phenotypic variation resulted from a strong genetic control. The continuous distribution of “fermentation capacity” in the population, suggests that this character is quantitative in nature. Phenotype distribution was also consistent with the phenotype being determined by several genes. Comparison of the parental strains with the segregant population revealed that only one segregant had a greater fermentation capacity than the parental 2029-C5 strain, whereas 16 segregants had a lower fermentative capacity than the 1782-B1 strain. Thus, the allelic combination responsible for the low-nitrogen requirement phenotype is rare. Conversely, the fermentative capacity of the segregants was lower than that of strain MTF1782, indicating that there are various negative alleles in the genomes of these two parental strains that can be combined.We generated a large population of F2 segregants (133), for the detection of combinations of several QTL explaining this complex phenotype. We overcame the difficulties associated with the genotyping of such a large population by adopting a bulk segregant analysis strategy. The most distant phenotypes were selected and combined in two pools of 15 segregants each. Each strain chosen for the two bulks the “low-nitrogen requirement” (LNR-bulk) and “high-nitrogen requirement” (HNR-bulk) bulks was characterized in greater detail by online fermentation monitoring. The segregants within each pool had similar fermentation profiles, whereas the two pools had contrasting fermentation profiles (Figure 3). The differences between the two bulks were most clearly visible during the onset of stationary phase (mid-fermentation at 50 hours of fermentation) and at the end of fermentation.

### Identification of the QTL region by bulk segregants analysis (BSA)

**Figure 4:**
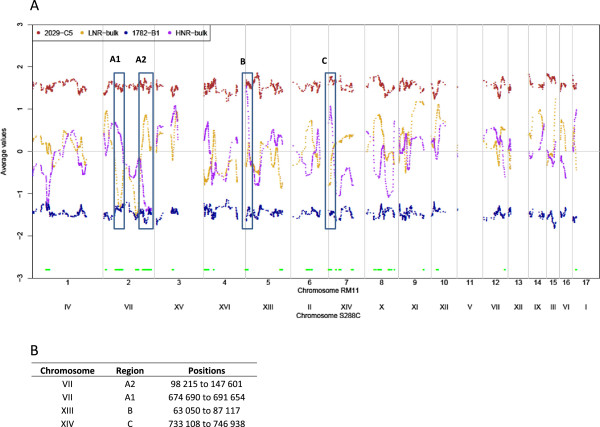
**Quantitative allele frequency measurement in DNA pools.** Genotyping of parental strains (2029-C5 in brown and 1782-B1 in deep purple), and two segregating pools with low (LNR orange) and high (HNR in purple) nitrogen requirements. The 2029-C5 allele enrichment of the pools is indicated by deviations above 0 and 1782-B1 allele enrichment is indicated by deviations below 0. Regions with significant differences in allelic frequencies between the two pools are indicated by light green dots. RM11 probes were positioned on the S288C genome by Blast. Four regions displaying large differences between the two pools were chosen for further analysis: **A1**, **A2**, **B** and **C**.

For the detection of genomic regions involved in the “fermentation capacity” trait, we hybridized genomic DNA from the two pools of 15 segregants and the two parental strains to an Agilent 8x15K custom isothermal array containing 6318 SNPs. The array was obtained from a comparison of the genomic sequences of P3-D5 and RM11, strains genetically similar to the 2029-C5 and 1782-B1 parental strains, respectively (see Methods). The hybridization signals for the 1900 significant oligonucleotides are plotted along the chromosomes for the two pools of segregants and the two parental strains (Figure 4A). There are large and significant (indicated by green spots) differences between the two bulks in several regions. For some regions, the maximum hybridization intensities indicate the almost exclusive presence of a single parental allele in one bulk. For example, this was observed once on the chromosome IV homologous to the S288C chromosome (1,163,169 to 1,214,335 bp), whereas the HNR-bulk displayed the same signal as the 1782-B1 parent, and three times on the chromosome VII homologous to the S288C chromosome (98,215 bp to 147,601 bp; 674,690 bp to 691,654 bp; 730,853 bp to 765,691 bp) whereas the HNR-bulk displayed the same signal as the 2029-C5 parent for the first two peaks and the signal of the 1782-B1 parent for the third peak. The hybridization profiles suggest that 23 regions differed significantly between the two bulks. However, the small population used for each bulk may have resulted in local variations in the hybridization signal, leading to the detection of false-positive peaks. We reduced this risk to obtain false positive by restricting arbitrarily our analysis to regions for which the differences between the hybridization signals obtained for the two bulks for each allelic probe were significant (as determined with a 20-SNP window *t*-test) and greater than half the difference between the parents. Given the high number of peaks, genomic regions exhibiting the highest differences between each bulk are likely to have the highest impact on the phenotype. The resulting four QTL regions were chosen for analysis (Figures 4A and 4B): two on the chromosome homologous to S288C chromosome VII, which were approximately 17 and 50 kb long (regions A1 and A2), one 24 kb-long region on the chromosome homologous to S288C chromosome XIII (region B) and one 14 kb-long region on the chromosome homologous to S288C chromosome XIV (region C).

For regions A1, B and C, the bulk with the low nitrogen requirement (LNR-bulk) contained the alleles of the parent with the opposite pattern of nitrogen requirement, whereas the bulk with the high nitrogen requirement (HNR-bulk) contained specific markers of the 2029-C5 parent. By contrast, the LNR-bulk and the HNR-bulk contained 2029-C5 and 1782-B1 alleles, respectively, in the A2 region. These results suggest that, for three of the four QTL regions identified, the allele increasing fermentation rate originates from the strain with the lowest fermentation capacity (1782-B1), whereas only one allele from the strain with the highest fermentative capacity (2029-C5) had a positive effect on fermentation kinetic at one locus.

### Characterization of polymorphism for the candidate genes

We examined the four QTL regions, to identify the best candidate genes (Additional file 2: Figures S2 and Additional file 3: Figure S3). We chose four genes involved in nitrogen metabolism: *MDS3*, *GCN1*, *ARG81*, and *BIO3. MDS3,* which is located in QTL region A2, encodes a component of the TOR pathway, and *GCN1,* from the same region, is a regulator Gcn2p kinase involved in general amino-acid control [45]. These two genes are involved in nitrogen sensing and signaling in response to nitrogen availability. *ARG81,* located in the QTL region B, is a relevant candidate gene because it encodes a transcription factor that regulates gene expression in response to arginine availability. The *BIO3* gene is located in QTL region C and encodes a protein involved in biotin synthesis.

**Figure 5:**
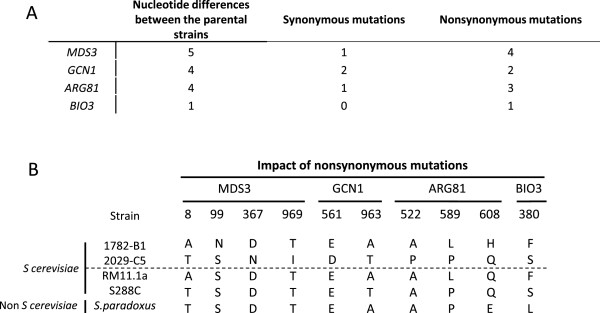
**Nucleotide sequence variation for the four candidate genes.** Nucleotide sequence variation between the two parental strains (2029-C5 and 1782-B1) **(A)**. Amino-acid differences between strains **(B)**.

We investigated whether polymorphism of the coding regions of these genes could account for phenotypic variation, by sequencing the four genes of the two parental strains (Figure 5A). We detected several nonsynonymous allelic variants (Figure 5B) that could potentially account for differences in nitrogen requirement. As phenotypic differences may also reflect differences in gene expression, we also compared the expression levels of these genes, as determined in a previous study [5]. No significant difference was found between MTF1782 and MTF2029 for the expression of these four genes, suggesting that the variations in expression were not involved.

### Functional analysis

**Figure 6:**
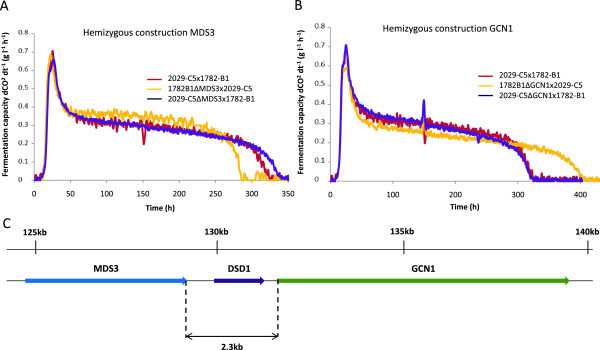
**Comparison of fermentation kinetics between hemizygous constructions.** Comparison between fermentation profiles for hemizygous construction. *MDS3* **(A)** and *GCN1* **(B)**. Localization of the two genes in the QTL region on chromosome VII (A2 region) **(C)**.

**Figure 7:**
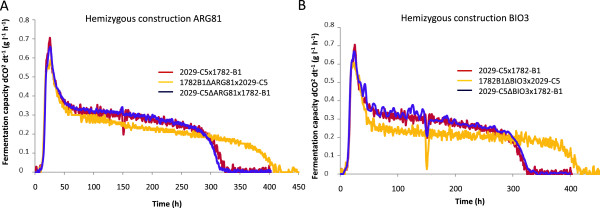
**Comparison of fermentation kinetics between hemizygous constructions.** Comparison between fermentation profiles for hemizygous construction. *ARG81* **(A)** and *BIO3* **(B)**.

For each candidate gene, we constructed two hemizygotic strains, by crossing one parent bearing an inactivated form of the gene with the other parent containing a functional form. Each hemizygote was phenotyped on nitrogen-deficient medium (SM100) and the fermentation kinetics of the strains were compared (Figures 6 and 7).

*MDS3* was the only gene tested for which a hemizygous construction with an allele from strain MTF2029 (1782-B1Δx2029-C5) resulted in a higher fermentation capacity than a hemizygous construction with an allele from MTF1782 (Figure 6A). The difference between the two hemizygous constructions corresponded to about 7% of the difference between the two parental strains in stationary phase (70 hours into the fermentation) and 15% of total fermentation time. *MDS3* was also the only gene for which a hemizygous strain had a higher fermentation capacity than the hybrid. The simultaneous expression of the two alleles in the hybrid thus has a negative effect on fermentation, resulting in a lower fermentation capacity than the expression of the allele originating from MTF2029 alone. Studies of hemizygotes for the *GCN1* gene showed that the allele from parent 1782-B1 conferred a higher fermentation capacity than the allele from 2029-C5 (Figure 6B). Total fermentation time differed by about 20% between these two hemizygotes. The effects of the *GCN1* and *MDS3* genes were interesting, because both these genes are located in QTL region A2. Indeed, they are separated by 2364 nucleotides but have opposite effects on fermentative capacity on nitrogen-deficient medium (Figure 6C). The determination of a possible complex genetic interaction between alleles *MDS3* and *GCN1* requires further characterization of this QTL structure. The reciprocal hemizygosity tests performed for each single gene did not permit to assess the combined effects of alleles. In the parental strain 2029-C5, the *MDS3* gene has a positive impact on this phenotype, whereas the *GCN1* gene has a negative effect (Figures 6A and 6B). For the parental strain 1782-B1, phenotypic comparison and nucleotidic variation between different strains show that *MDS3* is a recessive allelic form and *GCN1* is a dominant allelic form.

The differences in fermentation kinetics between the two hemizygous constructions (Figure 7A) confirmed the involvement of *ARG81* in fermentation capacity. The allele from the parental strain 1782-B1 conferred a better fermentative performance than the allele from parental strain 2029-C5, with fermentation time about 29% shorter for the 1782-B1 allele. However, *BIO3* hemizygous constructions had the most significant effect on fermentation rate under nitrogen limitation conditions (Figure 7B). The difference between the two hemizygotes corresponded to about 20% of the difference between the parental strains for fermentation rate during stationary phase and total fermentation time. The hemizygous phenotypes of *ARG81* and *BIO3* were consistent with the reverse distribution of markers in these two QTL regions: i.e. the HNR-bulk contains specific markers of 2029-C5.

**Figure 8:**
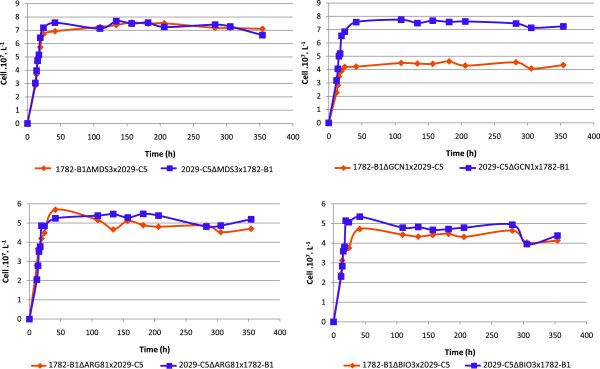
**Influence of each allele of the 4 candidate genes (** ***MDS3*** **: A,** ***GCN1*** **: B,** ***ARG81*** **: C,** ***BIO3*** **:D) on the total cell population.** The influence of allelic variation was evaluated by studying hemizygous constructions during fermentation in SM100 medium.

The total fermentation rate and the exponential growth phase were unaffected by the inactivation of the four genes, in all the constructions tested. A comparison of cell growth revealed no significant difference between the two hemizygous constructions for *MDS3*, *BIO3* and *ARG81* (Figure 8). For the *GCN1* gene, the growth profiles of the hemizygotes showed that the allele from 1782-B1 conferred higher levels of growth, resulting in a higher biomass, than the allele from 2029-C5.

These results confirm the involvement of these four genes in fermentative capacity in conditions of nitrogen limitation. The phenotypes obtained for the various hemizygotes remained very different from the phenotypes of the haploid parents. These findings highlight the complexity of this technological phenotype and demonstrate that the phenotype of the two haploid parents results from the allelic combinations of several genes, which may also interact.

## Discussion

In this study, we constructed and characterized a recombined F2 segregant population and used a BSA approach to identify genes involved in the variation of fermentation capacity under conditions of nitrogen limitation. This led to the detection of four genomic regions involved in “low” or “high” nitrogen requirement phenotypes. For three of these four regions, we were able to demonstrate that allelic variations of four genes had an impact on fermentation performance. Unexpectedly, for three of the four genes, the allele with a positive effect on fermentation rate originated from the strain with a low fermentation capacity in such conditions (1782-B1), rather from the strain with a high fermentation capacity, 2029-C5. This finding of multiple genes with positive effects in strains with a low fermentation capacity is consistent with the complex character of the trait, as suggested by the distribution of “nitrogen requirement” values in the recombined population. As we identified only one gene with a positive effect originating from 2029-C5, other genes not detected in this first analysis are probably involved in this phenotype, as suggested by the numerous peaks in the hybridization profile, corresponding to putative QTL regions. Our ability to detect more QTL is, of course, limited by the size of the segregant population, which also imposed constraints on the number of segregants per pool that we could analyze and by the phenotypic variation between the two selected bulks. The phenotyping of a larger population might lead to the identification of other genes involved in this complex phenotype.

One of the most interesting findings of this study is the organization of QTL region A2 into a complex architecture, with two alleles of *MDS3* and *GCN1* having opposite effects on the phenotype in the 2029-C5 strain. The *MDS3* allele has a positive effect on fermentation rate whereas the *GCN1* allele has a negative effect, and both genes are involved in mechanisms responding to nitrogen availability. The QTL was detected by hybridization as a region from 2029-C5 with a positive impact on fermentation rate (enriched in the LNR pool). As this QTL contained the two genes, the impact of *GCN1* allelic variation would be expected to be much weaker than that of *MDS3*. However, this supposed difference in power between the two genes was not observed in functional analysis based on reciprocal hemizygote tests, in which both alleles had a moderate to weak effect, suggesting that this assay may underestimate the effect of the *MDS3* allele. While the impact on the phenotype is low, *MDS3* and *GCN1* have antagonistic effects on the phenotype. Moreover these two genes have functional relationship. Physical and functional proximity of these genes suggest a possible interaction between alleles. This would be consistent with a moderate effect of the two alleles on the phenotype. This combination could provide a selective advantage to the strain and will justify the association of alleles with antagonistic effects. We could also suppose that this phenotype is the result of interaction with other genes located in other regions. More detailed characterization of the genes will be required to determine the impact of allelic variation. Three of the four genes identified as candidate genes in this study are directly involved in nitrogen metabolism, in sensing and signaling or regulating gene expression in response to nitrogen status. The remaining gene, *BIO3,* is involved in biotin metabolism, which is not directly linked to nitrogen metabolism.

These four genes harbor mutations predicted to result in amino-acid substitutions potentially affecting the activity of the encoded protein. All non-synonymous SNPs were analyzed by SIFT (Sorting Intolerant From Tolerant). This program is a tool based on sequence homology for predicting the probability that a mutation might be deleterious and affects the protein function [46]. Phylogenetic analysis (Additional file 4: Figure S4) showed that the two allelic forms of the different genes were relatively similar. Mds3p displays four nonsynonymous mutations with respect to the two parental strains, and two mutations are positioned in N-terminus protein. According to SIFT analysis, these mutations should not affect the protein function in a deleterious manner. Phylogenetic analysis suggested that two of the variants present in the 2029-C5 parental strain were ancestral whereas the other two were ancestral in the 1782-B1 strain (Figure 5B). For Gcn1p, phylogenetic analysis showed that the two nonsynonymous mutations in 1782-B1 corresponded to the ancestral form of the allele (Figure 5B). The second mutation is particularly interesting because it affects an Armadillo-like helical domain of the protein critical for its spatial conformation [47]. In this case, SIFT analysis indicate that the two mutations may impact the protein function. Comparative sequence analysis of the Arg81p protein showed that the two parents each have one mutation with respect to the ancestral allelic form (Figure 5B). The Bio3p protein displays one nonsynonymous substitution, for which it is difficult to confirm the ancestral allelic form. This substitution is located in a pyridoxal phosphate-dependent transferase domain of the protein. For ARG81 and BIO3, SIFT analysis did not reveal possible deleterious mutation.

For the four genes studied, the impact of the mutations identified on the activity of the proteins was unknown and difficult to infer from our data. *MDS3* is involved in the TOR signaling pathway and was shown to function as a positive regulator acting on *TAP42*[48]. TOR senses the nutrient status of the cell (specifically amino acids) and coordinates the cellular response through the control of protein synthesis and ribosome biogenesis, which are stimulated when TOR is active, whereas growth stops and stress response are triggered by nitrogen starvation and TOR inactivation. A previous transcriptomic comparison of the parental strains revealed differences in gene expression, such as higher levels of ribosomal gene expression in MTF2029 and of stress gene expression and protein degradation in MTF1782, consistent with a higher level of TOR pathway activity in the MTF2029 strain compared to MTF1782 [5]. *MDS3* expression did not differ significantly between the two strains but, as *MDS3* activates the TOR pathway, the variations of gene expression observed are consistent with higher activity of Mds3p in MTF2029. Moreover, this result is reminiscent of the impact of variation of the *RIM15* gene, encoding a TOR-controlled PAS kinase, on the fermentation rate of sake yeasts [13]. This previous study showed that changes in *RIM15* function prevented the entry of the cells into quiescence during starvation and led to the maintenance of high rates of glycolysis. Indeed, higher levels of *MDS3* activity could modulate TOR activity but the mechanisms by which such an increase in TOR activity could lead to an increase in fermentation rate are unclear. Studies suggest that TOR can impact glycolytic flux by a modulation of the PKA activity through different intermediates such a Gcn4p [49], FGM pathway [50] or ammonium permeases which act as sensor [51].

*GCN1* is known to be a positive regulator of general amino-acid control (GAAC) [45]. It is required for the activity of *GCN2,* which triggers the preferential translation of mRNA encoding the *GCN4* transcription factor, which controls the expression of genes encoding proteins involved in amino-acid biosynthesis [45]. Gcn1p forms a complex with Gcn20p. This complex is not required for *GCN2* activation, but increases the phosphorylation of eIF2α by Gcn2p [45]. However, *GCN2* regulation is not thought to be functional in nitrogen-starved cells [52]. Two mutations of the *GCN1* gene can be used to distinguish between the two parental allelic forms. One of these mutations affects an Armadillo repeat domain involved in the spatial conformation of this protein. It remains unclear how variations affecting Gcn1p causes changes in fermentation rate. It is thought that changes in the conformation of the protein may influence the formation of the Gcn1/Gcn20 complex and *GCN2* activation. A perturbation of *GNC2* activation could impact the activation of *GCN4* and intracellular amino-acid pools. *ARG81* is a transcription factor included in a protein complex (Arg81p, Arg80p, Mcm1p, Arg82p), controlling the expression of genes involved in the anabolism and catabolism of arginine [53]. Indeed, mutations in *ARG81* gene may lead to perturb the arginine metabolism and change the cellular pool of nitrogenous compounds [54]. It is not possible to infer the impact of the differences between the two *ARG81* forms on protein activity. However, it is possible that an increase in *ARG81* activity might lead to changes to the cellular amino-acid pool, with an impact on protein synthesis or nitrogen signaling.

*BIO3* encodes a DAPA aminotransferase involved in biotin biosynthesis [55]. The *BIO3* gene is not directly connected to nitrogen metabolism. However, biotin is involved in carboxylation reactions, some of which are involved in nitrogen metabolism, such as the reactions catalyzed by urea carboxylase or pyruvate carboxylase (generating oxaloacetate, a precursor of a-ketoglutarate and aspartic acid). Changes in biotin availability might therefore have an effect on the equilibrium of the nitrogen pool. Alternatively, its is notable that *BIO3* uses SAM (S-Adenosyl methionine) as substrate containing a nitrogen group and its activity might impact on SAM pool which is involved in other cellular metabolisms. The only mutation found in this gene affected the pyridoxal phosphate-dependent transferase domain. This domain is involved in key functions, such as the transfer of nitrogenous groups. Mutations affecting this domain may affect the concentration of biotin, which is required by the cell.

## Conclusion

In conclusion, we show here that fermentation performances in conditions of nitrogen limitation result from the cumulative effects of multiple alleles, suggesting that this phenotype is highly complex. This QTL study led to the identification of three candidate genes with functions associated with the regulation of nitrogen metabolisms, nitrogen sensing or signaling: *GCN1*, *ARG81* and *MDS3*. These findings highlight the role of nitrogen signaling in the control of glycolytic flux in nitrogen starvation and support the hypothesis that the TOR pathway plays a key role in controlling fermentation capacity in nitrogen-starved cells, consistent with previous observations [56]. These finding are consistent with the previous data indicating that the two strains did not have differences in protein synthesis capacity in such starved conditions. Additional studies are required to identify the precise mechanisms by which variations of TOR signaling modulate fermentation flux.

## Methods

### Construction of the parental strains

We used two enological *Saccharomyces cerevisiae* strains in this study: MTF2029 and MTF1782. The nitrogen requirements and phenotypic characteristics of these two strains were determined in a previous study [5]. Both these strains are homothallic *HO/HO* diploids. The *HO* gene was disrupted in both strains, to obtain haploid clones for the crossing experiments. Yeasts were transformed, as described by Guldener *et al.*[57], with pUG6 (KANMX6 cassette) for MTF2029 and pAG25 (NATMX4 cassette) for MTF1782. For the disruption of one copy of the *HO* gene, we used the same primers for both parents. The two custom-made 60-mer primers used began with 40 nucleotides identical to the upstream or downstream region of the *HO* gene, followed by 20 nucleotides for amplification of the KANMX6 and NATMX4 cassettes (Forward primer: ATGCTTTCTGAAAACACGACTATTCTGATGGCTAACGGTGCTTCGTACGCTGCAGGTC and Reverse primer: TTAGCAGATGCGCGCACCTGCGTTGTTACC ACAACTCTTTAGTGGATCTGATATCACCTA). Sporulation was allowed to occur and we then dissected transformants displaying integration of the drug resistance cassette. We collected haploid *ho* spores, which we grew on YPD (yeast extract, peptone and dextrose) plates supplemented with the corresponding antibiotic (G418 at 200 μg/ml for MTF2029 and cloNAT at 200 μg/ml for MTF1782*)*. Two haploid *ho* spores were selected: 2029-C5 from MTF2029 and 1782-B1 from MTF1782. A zygote was created by crossing 2029-C5 (Mat α) and 1782-B1 (Mat a) and tested on YPD plates supplemented with G418 and cloNAT.

### Construction of the segregant population

We generated an F2 population of segregants, as a means of obtaining more recombinants. The F1 segregant population was obtained by tetrad dissection of the parental cross 2029-C5x1782-B1. An F2 diploid population was then constructed by randomly crossing F1 segregants from different asci. The F2 diploids were then allowed to sporulate and the spores were collected by tetrad dissection. The ploidy status of the F2 segregants was checked in a mating test [26].

### Fermentation conditions

Phenotypic parameters were measured in batch fermentations in a synthetic medium (SM) as described by Bely *et al.*[58], mimicking a natural must contain: glucose (200 g liter^−1^), malic acid (6 g liter^−1^), citric acid (6 g liter^−1^), MgSO_4_ (250 mg liter^−1^), KH_2_PO_4_ (750 mg liter^−1^), CaCl_2_ (155 mg liter^−1^), NaCl (200 g liter^−1^), K_2_SO_4_ (0,5 g liter^−1^), vitamins and oligoelements mixtures. SM100 and SM120 was supplemented respectively with a final concentration of 100 and 120 mg liter^−1^ assimilable nitrogen corresponding to ammonium salt and a mixture of 19 amino acids (L-proline, L-glutamine, L-arginine, L-tryptophan, L-alanine, L-glutamic, L-serine, L-threonine, L-leucine, L-aspartic acid, L-valine, L-phenylalanine, L-isoleucine, L-histidine, L-methionine, L-tyrosine, L-glycine, L-lysine and L-cysteine). Batch fermentations were performed in a 1.2-liter fermenter and microfermenter (300 ml), with mixing by a magnetic stirrer (500 rpm), and airlocks to maintain anaerobiosis. We assessed the fermentation performances of the parental strains and hemizygotes in SM100 at 24°C, for precultures and fermentations. We carried out segregant analysis at 28°C in SM120, to make it possible to identify differences in fermentation performance more rapidly.

### Measurement of phenotypic parameters

Two phenotypic measurements based on the CO_2_ released during fermentation were obtained. The first phenotypic measurement was obtained at the start of the stationary phase (89 hours into the fermentation on SM120). Microfermentations were not monitored and fermenter mass was assessed manually. This measurement made it possible to distinguish rapidly between strains on the basis of their fermentation capacity on entry into stationary phase. This parameter was strongly correlated with the fermentation performance of the strains on nitrogen-deficient medium.

We studied the kinetics of fermentation by online monitoring (fermentation batch in 1.2 liter). The amount of CO_2_ released during fermentation was calculated from automatic measurements (taken every 20 min) of fermenter mass [59]. This fermentation monitoring method was validated in a previous study [60]. The rate of CO_2_ production was calculated by polynomial smoothing of the last 10 measurements of fermenter weight loss. The many acquisitions of data for the mass and the precision of weighing (0.1 to 0.01 g) made it possible to calculate the rate of CO_2_ production with a high level of precision [58]. The high frequency of online measurements of CO_2_ production (one measurement every 20 minutes) made it possible to calculate the rates of CO_2_ production by sliding-window second-order polynomial fitting in a custom-developed Labview application. This measurement made it possible to determine the overall rate of fermentation during stationary phase and to compare segregants at the same stage of fermentation.

### Reciprocal hemizygosity analysis

This technique was used for the identification of allele-specific contributions to the phenotype [61]. We used the same primers for each candidate gene and the same transformation strategy as for the two parental strains. We inactivated one copy of the candidate gene with the hygromycin selection cassette. We then carried out a specific PCR to check that the gene was indeed inactivated. The primers used for cassette integration are indicated in the supplementary data (Additional file 5: Figure S5). The contribution of the alleles to the phenotype was analyzed by comparing fermentation kinetics in SM100 (1.2-liter fermenters).

### Microarray design

For the detection of genetic variation in the segregant population, we designed a DNA microarray with isothermal-melting probes, as described by Gresham *et al.*[62]. The genome sequences of *Saccharomyces cerevisiae* RM11 and *Saccharomyces cerevisiae* P3-D5 (related to MTF1782 and MTF2029, respectively) were used to construct a set of 6,318 pairs of isothermal probes. Biallelic positions at least 100 bp apart were chosen. We also included 300 replicates and 1000 invariant control probes in the array. The primers were designed with primer3 [63], to ensure hybridization at 50°C. The array design is available on GEO under the accession number GPL18217.

### DNA extraction, labeling and hybridization conditions

For bulk segregant analysis, we selected two pools of 15 segregants each. The first pool contained the 15 strains with the best fermentation performances in nitrogen deficiency conditions and the second pool contained the 15 segregants with the poorest fermentation performance in such conditions. Each pool was treated separately. For each segregant, a culture in 30 ml of YEPD at 28°C was prepared and the number of yeast cells present in the culture was determined by counting with an electronic particle counter (Multisizer 3 counter; Beckman Coulter). For each pool, we isolated genomic DNA from all the segregants, mixed in equal proportions (5 x 10^9^ cells for each segregant), on Qiagen Genomic-tip 100/G columns. We also prepared genomic DNA from the parental strains, beginning with the same number of cells. For labeling and hybridization conditions, we used a modified version of the protocol described by Gresham *et al.*[62]. Genomic DNA was digested, fragmented by sonication and purified with a Qiagen Purification PCR kit. We labeled 1 μg of fragmented genomic DNA with Cy3 (for the two parental strains and the two bulks) or Cy5 (for a mixture of the DNA from the two parental strains) and carried out Agilent oligonucleotide array-based CGH for genomic DNA analysis, in accordance with the manufacturer's instructions (ref G4410-90010). Microarrays were hybridized at 59°C for 17 hours, then washed by immersion in two chip wash buffers: a low-stringency buffer (CGHmix2), followed by a high-stringency buffer (CGHmix1). Microarrays were scanned with a Genepix 4000B scanner (Axon Instruments Inc.).

### Statistical analysis

Statistical analysis was performed with R software, version 2.15.2 [64]. The phenotype heritability H^2^ was calculated as previously described [65], i.e. H^2^ = ((Var_seg_ – Var_env_)/Var_seg_) x100, where Var_env_ is the pooled variance among parental measurement and Var_seg_ is the variance among phenotype values for the segregants. Transgressive segregation was defined as in [65, 66] by the number of segregants whose phenotype level lay at least 2σ higher than the mean phenotype level of the higher parent or 2σ lower than the mean phenotype level of the lower parent; σ is the pooled standard deviation of the parents.

The Agilent 8x15K array was imported into R software with the limma package [67]. For each probe of each block, the log2 (green signal/red signal) ratio was calculated and centered. The red signal (Cy5) corresponded to the reference signal and the green signal (Cy3) corresponded to the signal for each bulk and each parent. Differences between log ratios corresponding to the P3-D5 and RM11 alleles at biallelic loci were calculated and normalized, such that the parental strain MTF2029 had a mean value of 1.5 and the parental strain MTF1782 had a mean value of −1.5. This criterion was used to analyze the differences between bulks. A first selection of probes was performed by carrying out a one-tailed *t* test based on parental strain criteria. Approximately 1900 specific probes were selected after Benjamini-Hochberg correction for multiple testing [68] (adjusted *p*-values < 0.05). We tried to identify allelic positions differentiating the HNR-bulk from the LNR-bulk for these 1900 specific probes, by carrying out *t* tests on sliding sliding windows (20 probes per window) along the length of the genome. Benjamini-Hochberg correction of the *p*-values was performed. Only probes with adjusted *p*-values < 0.01 at positions at which differences between bulks were greater than one third the differences between the parents were retained (328 probes investigated). The complete array dataset (raw and processed data) is available from the Gene Expression Omnibus database under accession number GSE54389.

### Sequence analysis

For each candidate gene, we compared gene and protein sequences, to identify nonsynonymous mutations distinguishing between the two parental alleles. The polymorphic change was analyzed by SIFT (Sorting Intolerant From Tolerant) [46]. For each allelic form, we analyzed its distribution in the genomes of strains available from the *Saccharomyces* Genome Database (SGD, http://www.yeastgenome.org) and strains from the *Saccharomyces* Genome Resequencing Project (SGRP, 42 strains) [69, 70]. Phylogenies were inferred with MEGA [71], by the maximum likelihood method, based on the Kimura two-parameter model [72]. The trees with the highest log likelihood are shown. The trees are drawn to scale, with branch lengths proportional to the number of substitutions per site.

## Electronic supplementary material

Additional file 1: Figure S1: Fermentation profiles in nitrogen-deficient medium (SM100), at 24°C, for the two parental strains (2029-C5 and 1782-B1) and for the hybrid strain 2029-C5x1782-B1. (PDF 254 KB)

Additional file 2: Figure S2: QTL region map for the region A1 (A) and the region A2 (B). For the region A2, the map is divided into two parts. (PDF 136 KB)

Additional file 3: Figure S3: QTL region map, for the region B (A) and the region C (B). (PDF 122 KB)

Additional file 4: Figure S4: Molecular phylogenetic tree for the four candidate genes *(MDS3*: A, *GCN1*: B, *ARG81*: C, *BIO3*:D). Evolutionary history was inferred by the maximum likelihood method, based on the Kimura 2-parameter model and using 43 nucleotide sequences from the available genome sequences [69] (SGRP2), 70 (SGRP1), [72]. (PDF 225 KB)

Additional file 5: Figure S5: Primers used for cassette integration for gene inactivation in the parental strains. (PDF 170 KB)
