# Supplementary figures and images for: A genetic approach of wine yeast fermentation capacity in nitrogen-starvation reveals the key role of nitrogen signaling
Source: BMC Genomics. 2014 Jun 19;15(1):495. doi: 10.1186/1471-2164-15-495 (PMC4073503; doi:10.1186/1471-2164-15-495)

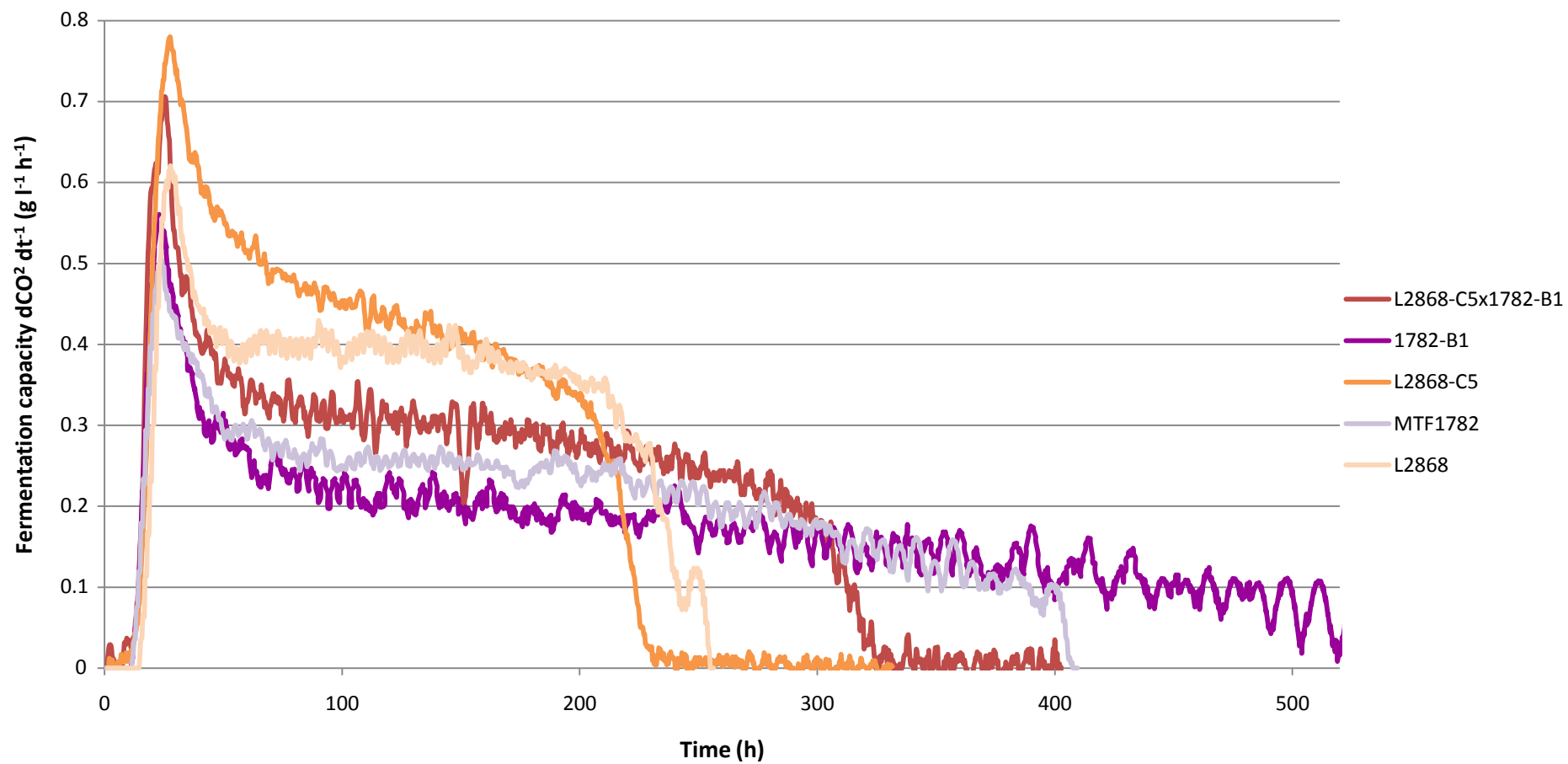

Supplement: Supplementary file 1 — Additional file 1: Figure S1: Fermentation profiles in nitrogen-deficient medium (SM100), at 24°C, for the two parental strains (2029-C5 and 1782-B1) and for the hybrid strain 2029-C5x1782-B1. (PDF 254 KB) [file 12864_2014_6161_MOESM1_ESM.pdf]

A

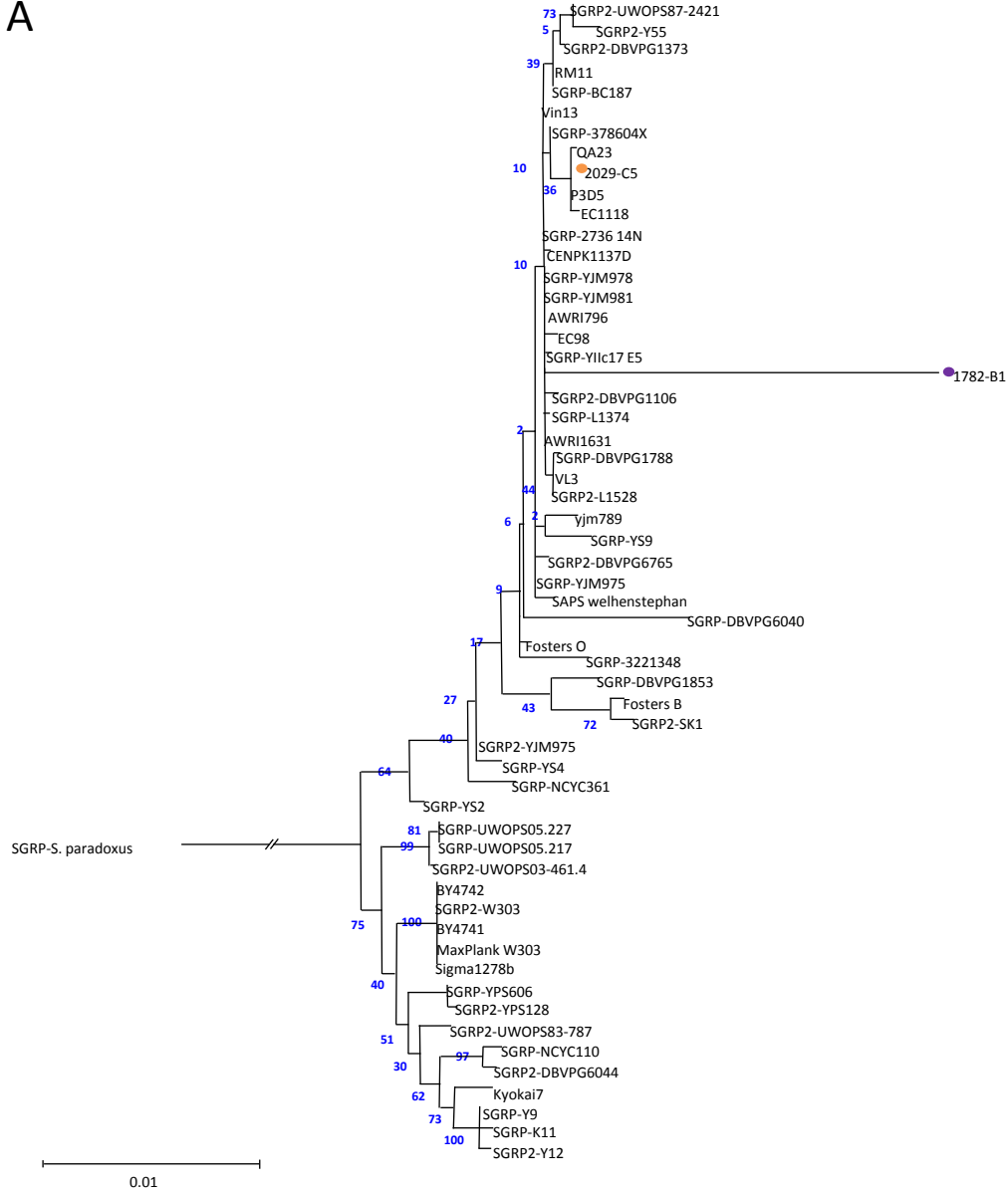

B

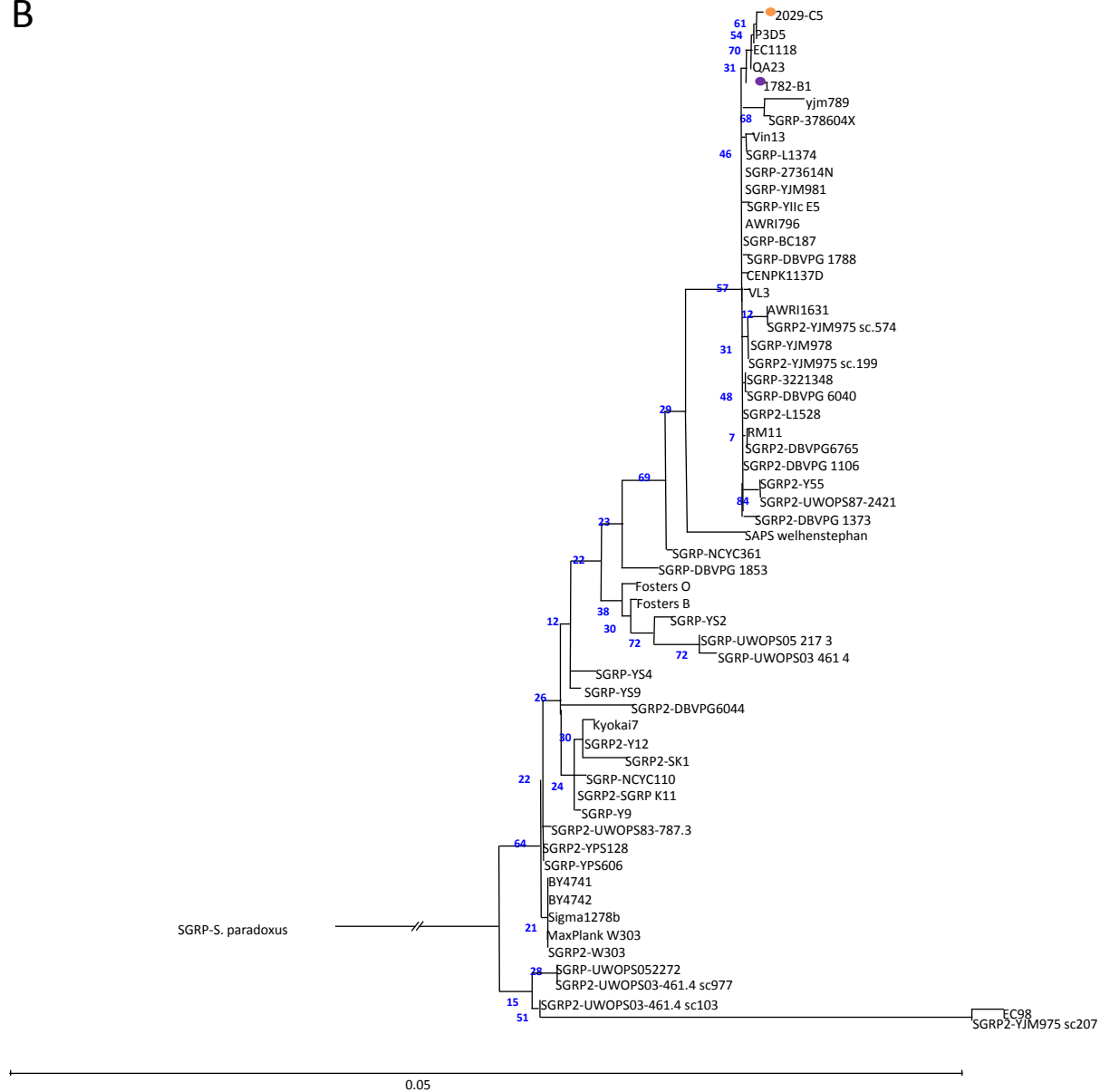

C

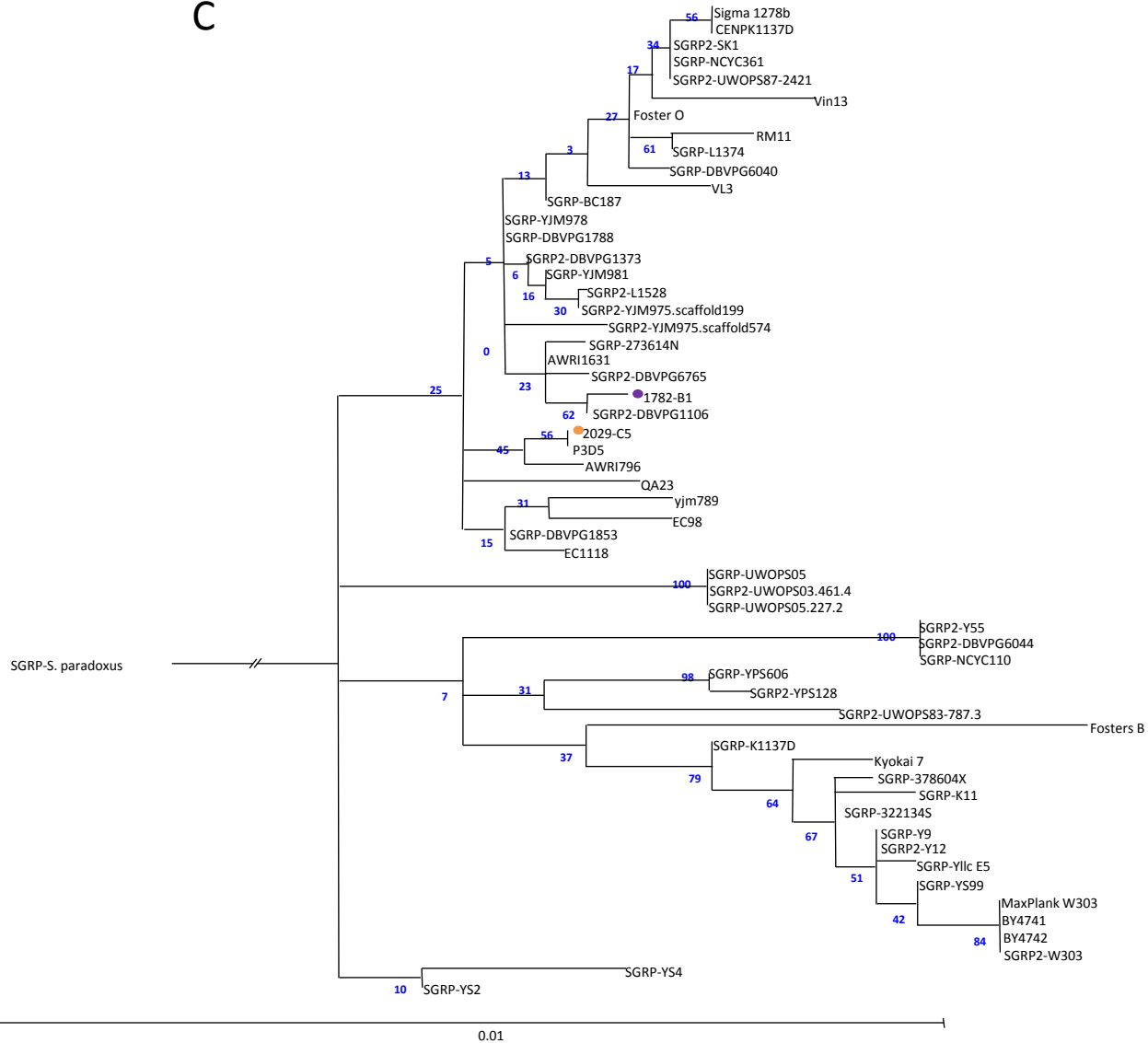

D

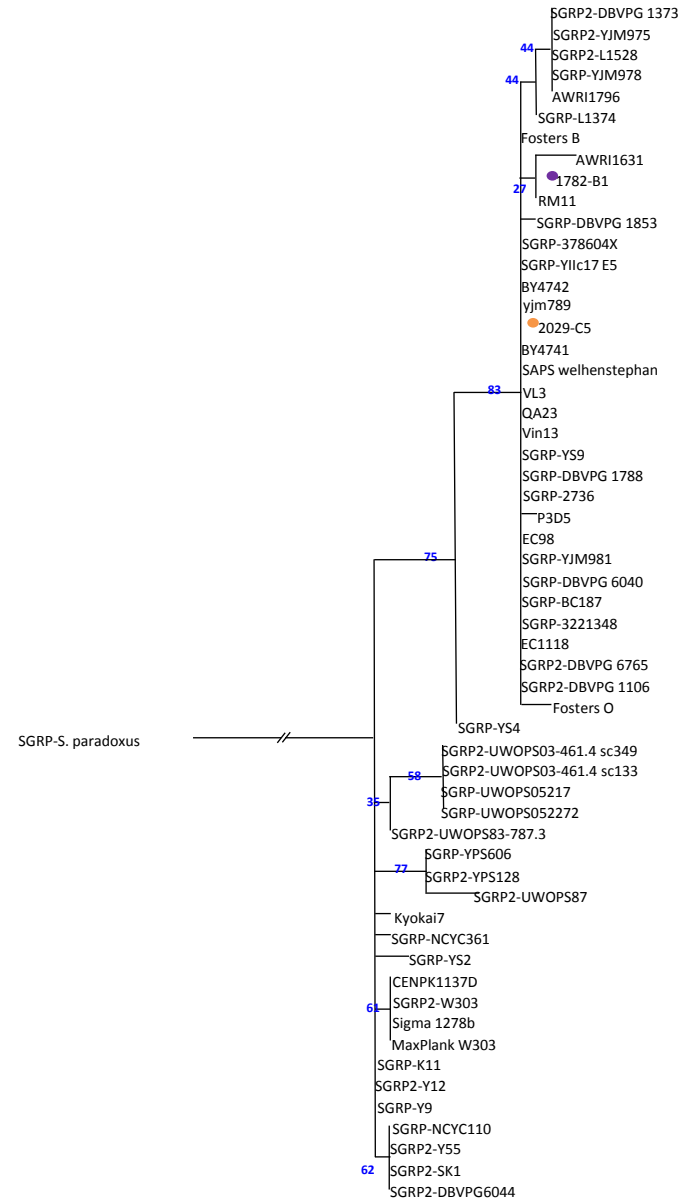

0.05

Supplement: Supplementary file 4 — Additional file 4: Figure S4: Molecular phylogenetic tree for the four candidate genes (MDS3: A, GCN1: B, ARG81: C, BIO3:D). Evolutionary history was inferred by the maximum likelihood method, based on the Kimura 2-parameter model and using 43 nucleotide sequences from the available genome sequences [69] (SGRP2), 70 (SGRP1), [72]. (PDF 225 KB) [file 12864_2014_6161_MOESM4_ESM.pdf]
