# Supplementary material for: A genetic approach of wine yeast fermentation capacity in nitrogen-starvation reveals the key role of nitrogen signaling
Source: BMC Genomics. 2014 Jun 19;15(1):495. doi: 10.1186/1471-2164-15-495 (PMC4073503; doi:10.1186/1471-2164-15-495)
Supplement: Supplementary file 2 — Additional file 2: Figure S2: QTL region map for the region A1 (A) and the region A2 (B). For the region A2, the map is divided into two parts. (PDF 136 KB) [file 12864_2014_6161_MOESM2_ESM.pdf]

A

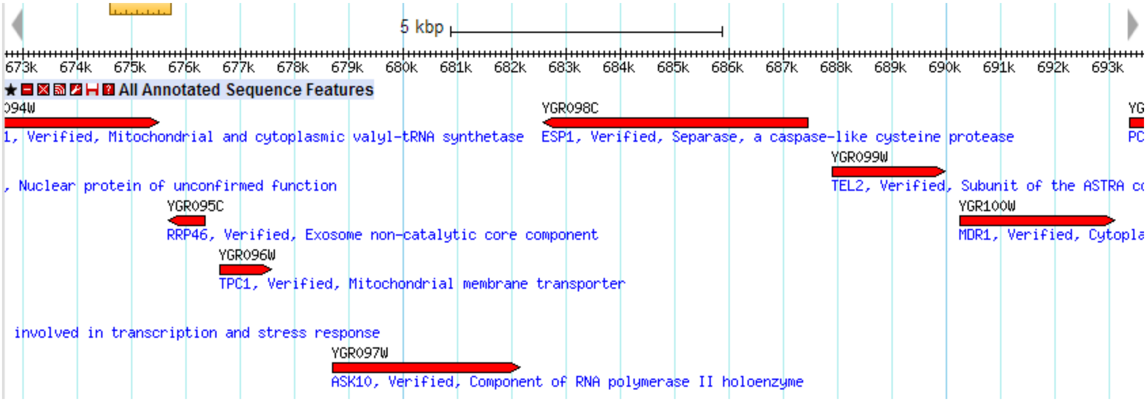

QTL region on chromosome VII (674 690 to 691 654)

B

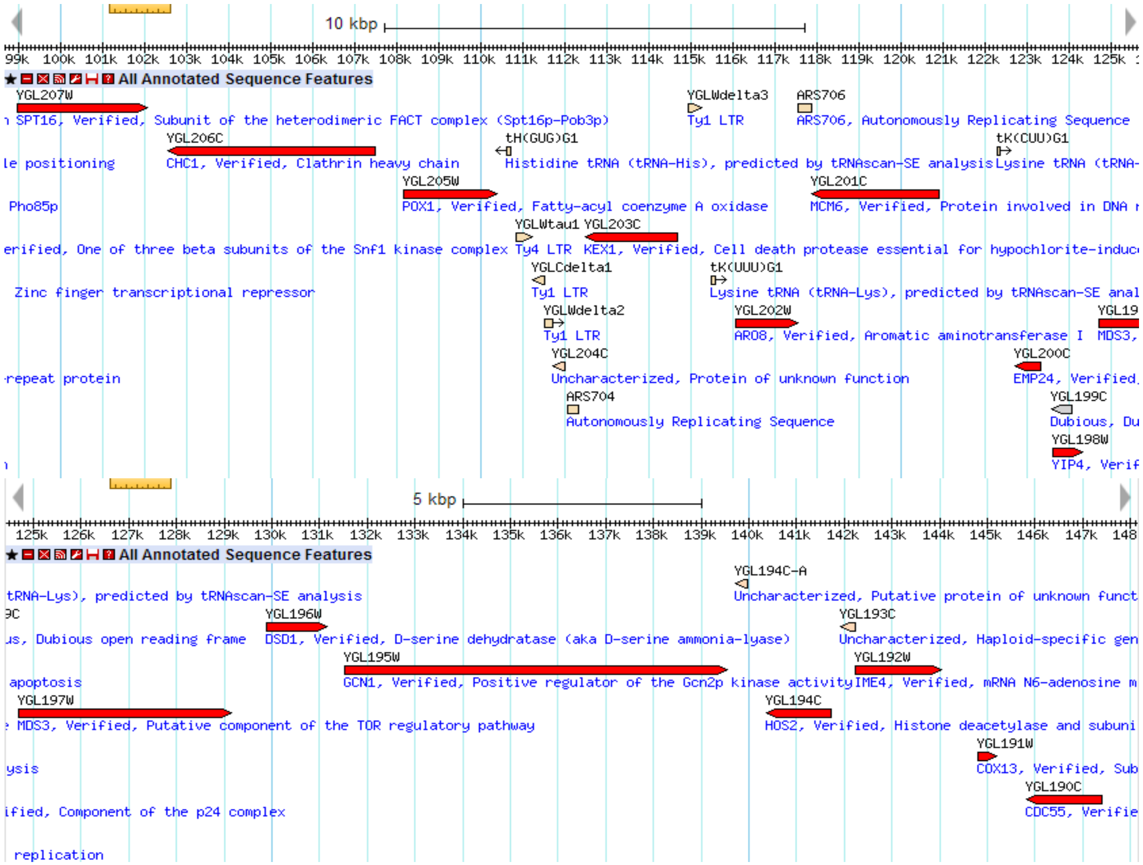

QTL region on chromosome VII (98 215 to 147 601)
