# Supplementary material for: A genetic approach of wine yeast fermentation capacity in nitrogen-starvation reveals the key role of nitrogen signaling
Source: BMC Genomics. 2014 Jun 19;15(1):495. doi: 10.1186/1471-2164-15-495 (PMC4073503; doi:10.1186/1471-2164-15-495)
Supplement: Supplementary file 3 — Additional file 3: Figure S3: QTL region map, for the region B (A) and the region C (B). (PDF 122 KB) [file 12864_2014_6161_MOESM3_ESM.pdf]

A

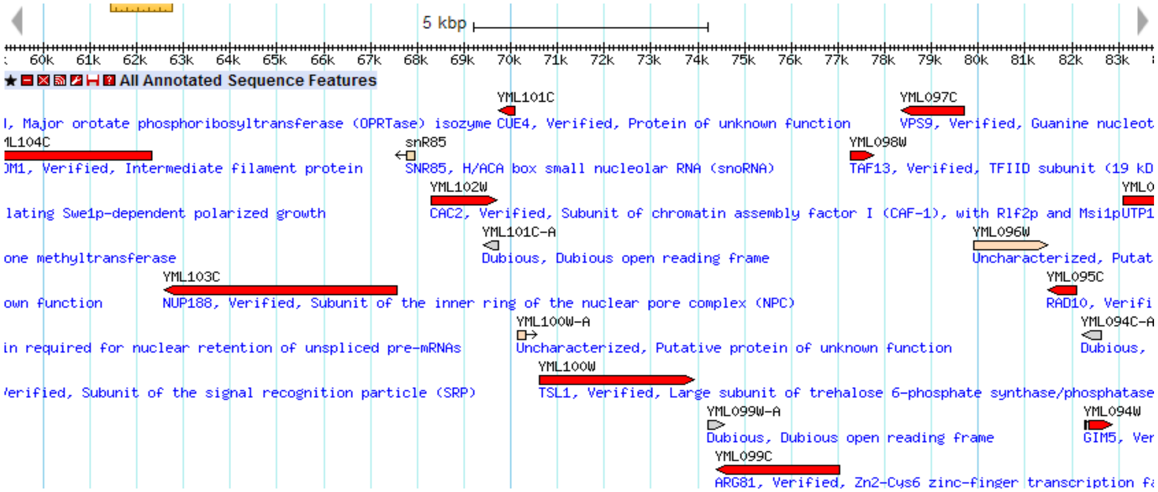

QTL region on chromosome XIII (63 050 to 87 117)

B

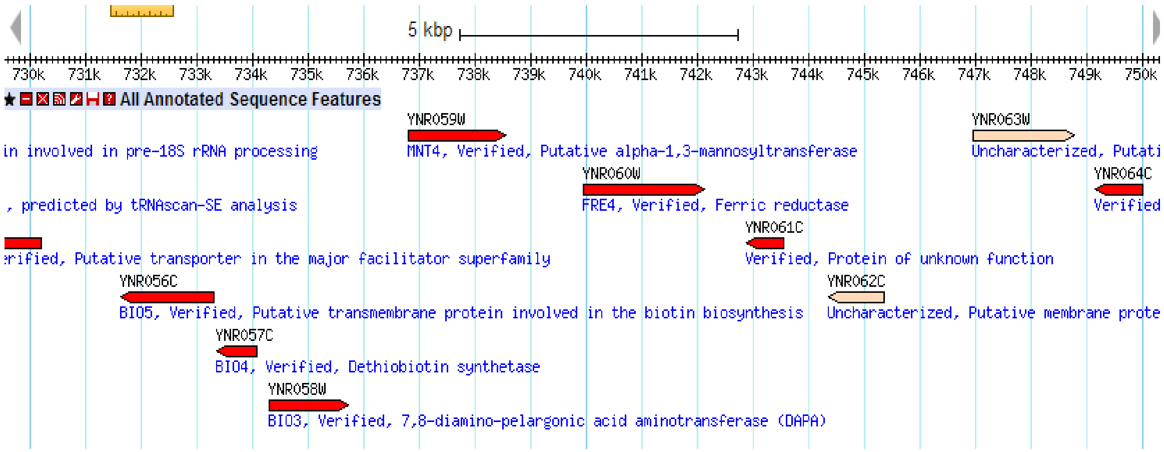

QTL region on chromosome XIV (733 108 to 746 938)
