# Supplementary material for: A genetic approach of wine yeast fermentation capacity in nitrogen-starvation reveals the key role of nitrogen signaling
Source: BMC Genomics. 2014 Jun 19;15(1):495. doi: 10.1186/1471-2164-15-495 (PMC4073503; doi:10.1186/1471-2164-15-495)
Supplement: Supplementary file 5 — Additional file 5: Figure S5: Primers used for cassette integration for gene inactivation in the parental strains. (PDF 170 KB) [file 12864_2014_6161_MOESM5_ESM.pdf]

Chr	Location (kb)	PCR primers	Primer sequences (5' -> 3')
VII	124.126	MDS3delF	CCAAAGGACAAGTAACAATCTGCACCTGCTTTAGCGTAGTTTCGTACGCTGCAGGTCGAC
VII		MDS3delR	TTGTACACTGGGTGAATAATAATCATCAGTCTTGGTACCCGCATAGGCCACTAGTGGATCTG
VII	131.182	GCN1delF	GTGATGACAGCTATCTTAAACTGGGAAGATATATCCCCTGTTCGTACGCTGCAGGTCGAC
VII		GCN1delR	CGGAGTGCAGAGCTGGTTGCCTCGATAATAACTGAAGTGCGCATAGGCCACTAGTGGATCTG
XIII	76.77	ARG81delF	CCTTGCGAAAAGCATCTCTTTTAGATTTGATGCTTTAGTGTTCGTACGCTGCAGGTCGAC
XIII		ARG81delR	CTGGTAAAGTTTATCATTGGCACTGGCTGCTTTATTAGGCATAGGCCACTAGTGGATCTG
XIV	734.735	BIO3delF	GGAGGATGTCAACAGCGCAATTCGTTTTCTATTGCTAGGATTCGTACGCTGCAGGTCGAC
XIV		BIO3delR	GAAATAAGAATTCCTTGGCAAATGACTTGATGAGATCGGCATAGGCCACTAGTGGATCTG
